# Sperm DNA methylome abnormalities occur both pre- and post-treatment in men with Hodgkin disease and testicular cancer

**DOI:** 10.1186/s13148-022-01417-1

**Published:** 2023-01-07

**Authors:** Donovan Chan, Kathleen Oros Klein, Antoni Riera-Escamilla, Csilla Krausz, Cristian O’Flaherty, Peter Chan, Bernard Robaire, Jacquetta M. Trasler

**Affiliations:** 1 Research Institute of the McGill University Health Centre, 1001 Décarie Boul. Block E, Montréal, QC Canada; 2 Lady Davis Institute for Medical Research, Jewish General Hospital, Montréal, QC Canada; 3 Andrology Department, Fundació Puigvert, Universitat Autònoma de Barcelona, Instituto de Investigaciones Biomédicas Sant Pau (IIB-Sant Pau), Barcelona, Catalonia Spain; 4 Department of Biomedical, Experimental and Clinical Sciences Mario Serio, University of Florence, Florence, Italy; 5 Department of Surgery, McGill University, Montréal, QC Canada; 6 Department of Pharmacology and Therapeutics, McGill University, Montréal, QC Canada; 7 Department of Urology, McGill University, Montréal, QC Canada; 8 Department of Obstetrics and Gynecology, McGill University, Montréal, QC Canada; 9 Departments of Pediatrics and Human Genetics, McGill University, Montréal, QC Canada

**Keywords:** Sperm, DNA methylation, Chemotherapy, Testicular cancer, Hodgkin disease

## Abstract

**Background:**

Combination chemotherapy has contributed to increased survival from Hodgkin disease (HD) and testicular cancer (TC). However, questions concerning the quality of spermatozoa after treatment have arisen. While studies have shown evidence of DNA damage and aneuploidy in spermatozoa years following anticancer treatment, the sperm epigenome has received little attention. Our objectives here were to determine the impact of HD and TC, as well as their treatments, on sperm DNA methylation. Semen samples were collected from community controls (CC) and from men undergoing treatment for HD or TC, both before initiation of chemotherapy and at multiple times post-treatment. Sperm DNA methylation was assessed using genome-wide and locus-specific approaches.

**Results:**

Imprinted gene methylation was not affected in the sperm of HD or TC men, before or after treatment. Prior to treatment, using Illumina HumanMethylation450 BeadChip (450 K) arrays, a subset of 500 probes was able to distinguish sperm samples from TC, HD and CC subjects; differences between groups persisted post-treatment. Comparing altered sperm methylation between HD or TC patients versus CC men, twice as many sites were affected in TC versus HD men; for both groups, the most affected CpGs were hypomethylated. For TC patients, the promoter region of *GDF2* contained the largest region of differential methylation. To assess alterations in DNA methylation over time/post-chemotherapy, serial samples from individual patients were compared. With restriction landmark genome scanning and 450 K array analyses, some patients who underwent chemotherapy showed increased alterations in DNA methylation, up to 2 to 3 years post-treatment, when compared to the CC cohort. Similarly, a higher-resolution human sperm-specific assay that includes assessment of environmentally sensitive regions, or “dynamic sites,” also demonstrated persistently altered sperm DNA methylation in cancer patients post-treatment and suggested preferential susceptibility of “dynamic” CpG sites.

**Conclusions:**

Distinct sperm DNA methylation signatures were present pre-treatment in men with HD and TC and may help explain increases in birth defects reported in recent clinical studies. Epigenetic defects in spermatozoa of some cancer survivors were evident even up to 2 years post-treatment. Abnormalities in the sperm epigenome both pre- and post-chemotherapy may contribute to detrimental effects on future reproductive health.

**Supplementary Information:**

The online version contains supplementary material available at 10.1186/s13148-022-01417-1.

## Background

Therapeutic advances in the field of oncology have allowed for an increase in the number of cancer survivors. However, the treatments used for effectively curing patients of their disease do not target cancer cells alone, but also adversely affect normal cells. Male germ cells are particularly susceptible to anticancer drugs due to the ongoing division of spermatogonial stem cells and continued production of mature spermatozoa following puberty; any possible negative impacts to the sperm cell may have implications for the next generation. Of particular interest are the survivors of Hodgkin disease (HD) and testicular cancer (TC), since they are often diagnosed at a young age. With a greater than 95% 5-year survival rate of adolescents and young adults for these two diseases [1, 2], concerns about fertility and the possibility of parenthood are particularly relevant [3, 4].

There are a number of studies showing genetic defects in spermatozoa, as well as more recent evidence of epigenetic abnormalities, following chemotherapy treatments [5, 6]; with this come questions regarding the possible impact of exposed germ cells to future generations. In a previous study, we reported increases in aneuploidy in the spermatozoa of HD and TC patients [7]. In the same cohort, increased sperm DNA damage, altered chromatin quality and decreased DNA compaction were found [8–10]. In these studies, abnormalities were found prior to the onset of treatment and remained up to 24 months after the termination of chemotherapy. Interestingly, studies have demonstrated that the risk of fathering offspring with a major congenital abnormality is increased in men with a history of cancer, even if the children were conceived prior to diagnosis and treatment, and this increase does not seem to diminish even years after treatment was given [11–13]. Thus, it is possible that both the cancer and its treatment may adversely affect male germ cells.

Animal models can help separate out effects of anticancer drug treatment from those of the underlying cancers being treated in humans. In one study, in which male rats were treated using similar drug regimens to those used in human TC patients, survival of offspring sired from these rats was found to be significantly reduced [14]. As well, there were increased DNA damage [15] and altered gene expression [16] in germ cells post-treatment. In the same model, altered sperm DNA methylation was detected immediately after the termination of combination chemotherapy treatments [17]. These studies indicate that along with persistent genetic and/or DNA damage in sperm, anticancer drugs used in human TC can result in sperm epigenetic alterations, in a rodent model, where there were also detrimental effects of the paternal treatment on the offspring.

DNA methylation, the most well-studied epigenetic mark, involves the covalent addition of a methyl group onto the cytosine ring. This modification occurs at 80% of CpG dinucleotides in the human genome and is involved in a number of cellular processes including transcriptional repression, X-inactivation and chromatin stability. Proper establishment of genomic imprinting, the monoallelic expression of genes in a parent-of-origin manner, also involves DNA methylation, with key events of demethylation and remethylation occurring in developing germ cells. Imprinted genes have important roles in embryonic growth and development, placental function and postnatal behavior [18–21].

The importance of DNA methylation is underscored by the role this epigenetic modification plays in different disease states. Abnormal expression of imprinted genes has been implicated in several developmental disorders including the Beckwith–Wiedemann, Silver Russell and Angelman syndromes [22, 23]. Aberrant DNA methylation has also been implicated in human infertility but remains controversial; altered methylation of imprinted loci has been reported in some studies examining human spermatozoa from men presenting with infertility and decreased sperm counts [24, 25], while other found no such associations [26]. Furthermore, genome-wide hypomethylation, as well as abnormal methylation of oncogenes and/or tumor suppressor genes, are all  hallmarks of cancer progression [27, 28].

With roles in cancer, as well as evidence of DNA methylation alterations associated with infertility and intergenerational effects [29, 30], our aim was to assess the sperm DNA methylation patterns in a cohort of HD and TC patients as well as a cohort of community control subjects (CC). The present study was designed to investigate the impact of HD and TC, as well as their treatments, on sperm DNA methylation patterns. Using several approaches to study genome-wide and locus-specific DNA methylation, our goals were to determine 1) whether abnormal germ cell DNA methylation patterns were present in patients prior to initiation of chemotherapy and 2) if, within the same patient, patterns of sperm DNA methylation were altered as a result of their cancer treatment, as well as over time post-treatment.

## Results

Following data processing and quality control, DNA methylation data generated using the Illumina HumanMethylation 450 K BeadChip (450 K) were available for 7 CC, 7 HD and 6 TC patients. Table 1 provides patient characteristics of these subjects at baseline, prior to initiation of chemotherapy. No significant differences between the three cohorts were observed in any of the quantitative measures.

**Table 1:** Characteristics at baseline, prior to chemotherapy initiation, of the Montreal patient cohort

Measure	CC	HD	TC
*n*	7	7	6
Age (years)	27.00 ± 2.29	25.00 ± 1.72	28.83 ± 1.14
Smokers	0	1	1
Sperm Conc. (millions/mL)	31.29 ± 5.16	91.29 ± 28.79	44.50 ± 10.46
Fast + Slow % Motility	48.29 ± 10.89	44.43 ± 1-.24	49.50 ± 8.99
Treatment	N/A	ABVD	Non-seminoma—BEP

Mean ± SEM

*CC* community control, *HD* Hodgkin disease, *TC* testicular cancer, *N/A* not applicable, *ABVD* doxorubicin, bleomycin, vinblastine, dacarbazine, *BEP* bleomycin, etoposide, cisplatin

### Imprinted gene DNA methylation is not affected by cancer or chemotherapy treatments

**Fig. 1:**
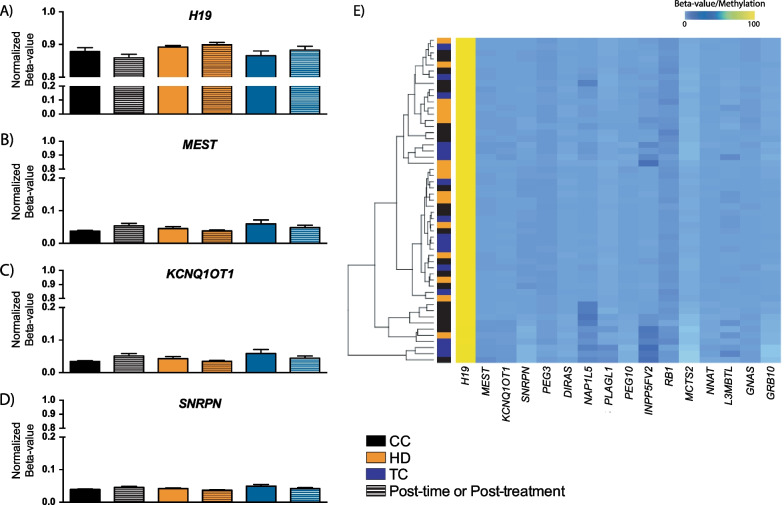
Sperm DNA methylation of imprinted genes is not affected by time (CC men), disease (HD or TC men) or treatments (HD or TC men). Methylation (beta-value) at the imprinting control regions of **A** *H19*, **B** *MEST*, **C** *KCNQ1OT1* and **D** *SNRPN*, examining samples at baseline (prior to initiation of treatment; solid bars) and post-time or post-treatment (hatched bars) times points. **E** Clustering of the average beta-values across the ICR of the different imprinted genes analyzed using the 450 K array

As key events of erasure and reestablishment of DNA methylation of imprinted genes occur in male germ cells during their development, we first examined this category of genes as a preliminary indication of the impact of HD and TC and their treatment on sperm. With the 450 K array, several probes are located within the germline imprinting control regions (ICRs) of numerous imprinted genes. We therefore extracted and averaged the beta-values/methylation of the probes within each ICR of individual imprinted genes from all the participants prior to and following treatment (Fig. 1 and Additional file 1: Table S1). We examined methylation of the ICRs of *H19*, *MEST*, *KCNQ1OT1* and *SNRPN*, genes previously reported to be altered in male infertility and/or regions known to have roles in imprinting disorders. For all four genes, there were no differences between the baseline samples for the CC, HD and TC groups (Fig. 1A-D, solid bars). Thus, for all pre-treatment sperm samples, high levels of methylation (> 85%) were found at the paternally methylated ICR of *H19*, while low levels (< 5%) were observed for the ICRs of the maternally methylated genes *MEST*, *KCNQ1OT1* and *SNRPN*. Similarly, at all times post-chemotherapy treatment, normal levels of methylation/beta-value (Fig. 1A–D, hatched bars) were found. Sperm DNA methylation levels of other imprinted genes found on the 450 K arrays also appeared normal and clustering based solely on imprinted gene methylation from all the samples was not able to distinguish the CC, HD and TC groups (Fig. 1E). In conclusion, imprinted gene methylation was not affected in the sperm of HD or TC patients either before or after anticancer drug therapy.

### Sperm DNA methylation differs between control and cancer groups prior to treatment

**Fig. 2:**
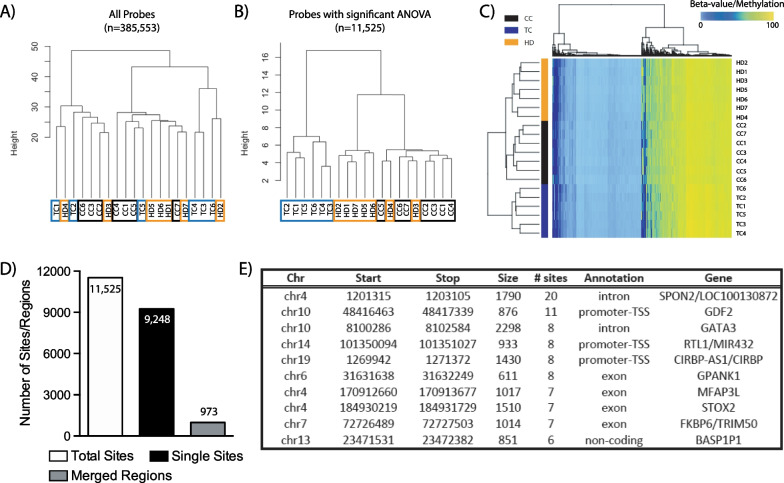
Sperm DNA methylation is capable distinguishing patients according to cancer diagnosis. Hierarchical cluster of 450 K probes at baseline, prior to chemotherapy treatment, for **A** all probes, **B** significant probes discovered by three-group ANOVA and **C** top 500 most significant probes. **D** Significant probes/sites discovered were merged to discover differentially methylated regions (DMRs), with **E** the top 10 DMRs containing the greatest number of merged probes/sites shown

Sperm samples were examined prior to chemotherapy to determine if the underlying cancer had an impact on the sperm DNA methylome. The baseline samples were first studied using the 450 K arrays. Of the over 450,000 probes found on the array, we removed probes with multiple mapping to locations, as well as probes containing single nucleotide polymorphisms at a minor allele frequency of greater than 1%. Hierarchical clustering of the beta-values/methylation for the remaining 385,553 probes was not able to differentiate the cancer cohorts from that of CC (Fig. 2A). Analysis of variance (ANOVA) was then applied to analyze the differences among the group means in the three different cohorts; this resulted in 11,525 probes with *p* values < 0.05 (Additional file 1: Table S2). Hierarchical clustering from these sites was able to distinguish TC patients and, to a lesser extent, the HD patients from CC (Fig. 2B). With clustering of the top 500 most significant probes, we were able to differentiate perfectly the three cohorts from one another (Fig. 2C). To ensure that these results did not happen by chance, we randomly selected the same number of probes found to be significant following the three-group ANOVA and observed no similar grouping of samples (Additional file 2: Fig. S1). These results demonstrate that a sperm DNA methylation signature, consisting of a subset of 450 K CpG sites, was able to distinguish HD and TC patients and CC subjects.

While probes represent individual CpG sites, we were interested to ascertain whether larger regions of altered methylation existed. To do so, we merged the significantly altered sites found within 1 kb of each other in order to obtain differentially methylated regions (DMRs). Of the 11,525 total sites discovered by ANOVA, a large majority of the sites remained isolated: 9248 single sites, representing 80.2% of the total sites (Fig. 2D). Despite this large proportion of isolated CpGs, DMRs were discovered, resulting in 973 merged regions. TopGO was used to look at gene ontology (GO) focusing on biological processes using the DMRs; the top 20 most significant terms are presented in Additional file 2: Fig. S2A, and all significant terms can be found in Additional file 3. Many terms pertaining to nervous system/synaptic signals as well as gene expression were discovered.

**Fig. 3:**
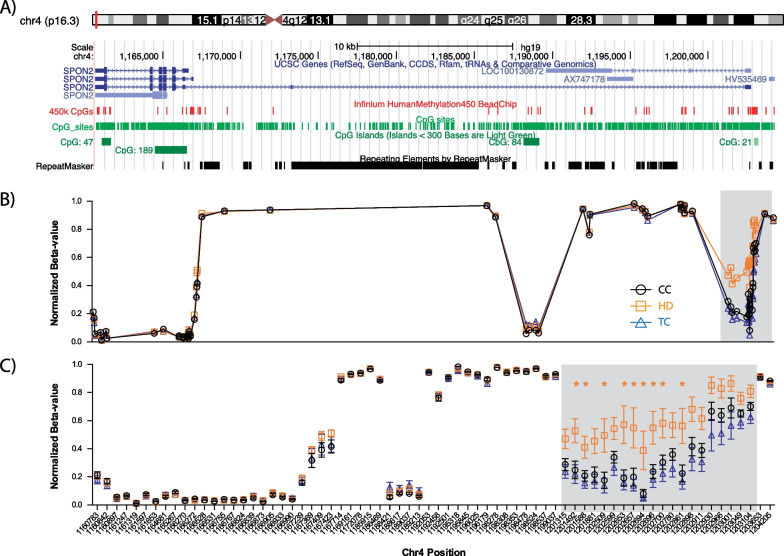
Schematic of the largest differentially methylated region (DMR) discovered following three-group ANOVA and post hoc Tukey’s test for HD. **A** UCSC genome browser view of the region encompassing the long non-coding RNA, LOC100130872, and the *SPON2* (Spondin 2) gene, with tracks showing location of 450 K probes (450 K CpGs) and all CpGs (CpG sites) within the region. Graphs of the average beta-value from each patient group are depicted, plotting the sites according to their **B** relative position within the genome and **C** separated to indicate SEM and significant CpG sites as discovered following post hoc testing. Highlighted areas in **B** and **C** indicate the DMR discovered following three-group ANOVA, while * indicates significant differences in post hoc Tukey’s testing. Mean ± SEM, *p < 0.05

The DMRs were then sorted according to the number of ANOVA significant sites, and the top 10 are listed (Fig. 2E). The large region found within the promoter and intronic elements of the transcript variant 3, of the spondin 2, *SPON2,* gene and the long non-coding RNA LOC100130872 (Fig. 3A) contains 20 sites (Fig. 3B, C, shaded box). Differential methylation can be observed between the three cohorts, with HD and TC patients possessing the highest and lowest averaged beta-value/methylation across the entire region, respectively. These results suggest that, beyond individual CpG sites, there are regions in the genome that are able to differentiate CC, HD and TC subjects using their sperm DNA methylation patterns.

DNA methylation of the same baseline sperm samples was examined with a second approach, restriction landmark genomic scanning (RLGS), a technique that examines approximately 3000 loci in the human genome. RLGS produces large two-dimensional gels where each locus is represented by a spot (Additional file 2: Fig. S3A); by overlapping gels, we are able to observe differences in methylation by the appearance (hypomethylation), disappearance (completely hypermethylation) or changes in spot intensity. Using a single CC patient as a reference (CC7), we compared all other sperm baseline samples from all other patients and found that many loci were altered in methylation (Additional file 2: Fig. S3B); however, no cancer-specific locus was observed when comparing all the HD and/or TC samples to the reference (i.e., locus specifically in cancer cohorts compared to CC). As RLGS analysis only allows loci differing in methylation between samples by more than 25% to be detected, the results suggest that large differences in methylation do not distinguish the 3 groups from one another using this technique.

### Hodgkin disease- and testicular cancer-specific sperm DNA methylation profiles

**Fig. 4:**
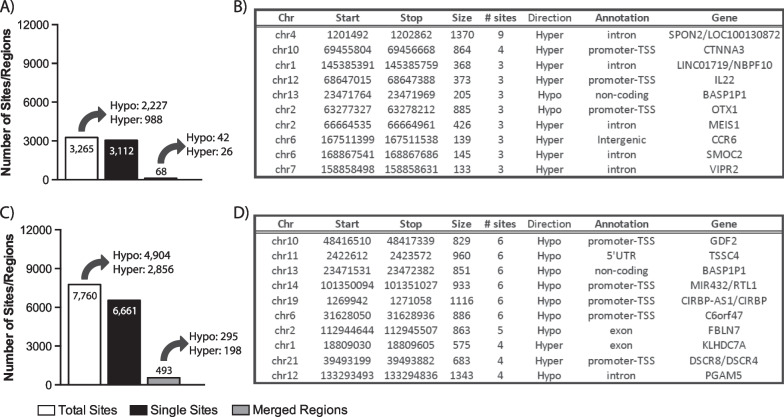
Discovery of differentially methylated regions following post hoc Tukey’s testing. Significant probes/sites discovered following post hoc Tukey’s testing between **A** HD vs. CC and **C** TC vs. CC patients were merged to discover differentially methylated regions (DMRs). A list of the top 10 DMRs found from **B** HD and **D** TC containing the greatest number of merged probes/sites

Next, we used the 450 K data to determine whether there were sites of differential methylation between specific cancer types (HD or TC) when compared with the CC group. Using post hoc Tukey’s testing, following our initial ANOVA analysis, a total of 3265 significantly altered probes were found between the HD and CC cohorts (Fig. 4A and Additional file 1: Table S3); more than twice the number of probes, 7760, were discovered for the TC cohort (Fig. 4C and Additional file 1: Table S4). For both cancer types, the majority of the differentially methylated sites demonstrated hypomethylation; 2277 sites (69.7%) showed lower methylation in the HD cohort, while 4904 sites (63.2%) were found in the TC cohort as compared to the CC.

Similar to the analyses above, cancer-specific DMRs were also identified. Once again, the majority of CpGs were single sites; 3112 and 6661 single sites (representing 93.3% and 84.2% of total significant sites) for the HD and TC groups, respectively, when compared to the CC group (Fig. 4A, C). When compared to the 68 DMRs for the HD cohort, greater than sevenfold more (493 DMRs) were found to be different between the TC and CC subjects. When looking at all DMRs together or with hyper- and hypomethylated DMRs separately, many biological processes related to immune system/response and development were found to be significantly enriched in the HD cohort (Additional file 2: Fig. S2B and Additional file 3). For the TC cohort, many GO terms related to development were found to be enriched (Additional file 2: Fig S2C and Additional file 3); interestingly, biological processes related to sexual/male reproduction were also discovered.

A list of the top 10 regions with the most CpG sites is shown for HD and TC (Fig. 4B and D, respectively). For HD DMRs, the largest region, containing the most sites (9 sites), was located in the *SPON2* gene, in the same region found through three-group ANOVA (Fig. 3). One large region, common to both the HD and TC cohorts, was found overlapping the non-coding RNA for the pseudogene brain-abundant, membrane-attached signal protein 1 pseudogene 1, *BASP1P1* (Additional file 2: Fig. S4A). All 450 K array probes within the vicinity of the pseudogene were found to be affected in at least one of the cancer cohorts, with lower levels of sperm DNA methylation observed in both compared with CC (Additional file 2: Figs. S4B and S4C).

**Fig. 5:**
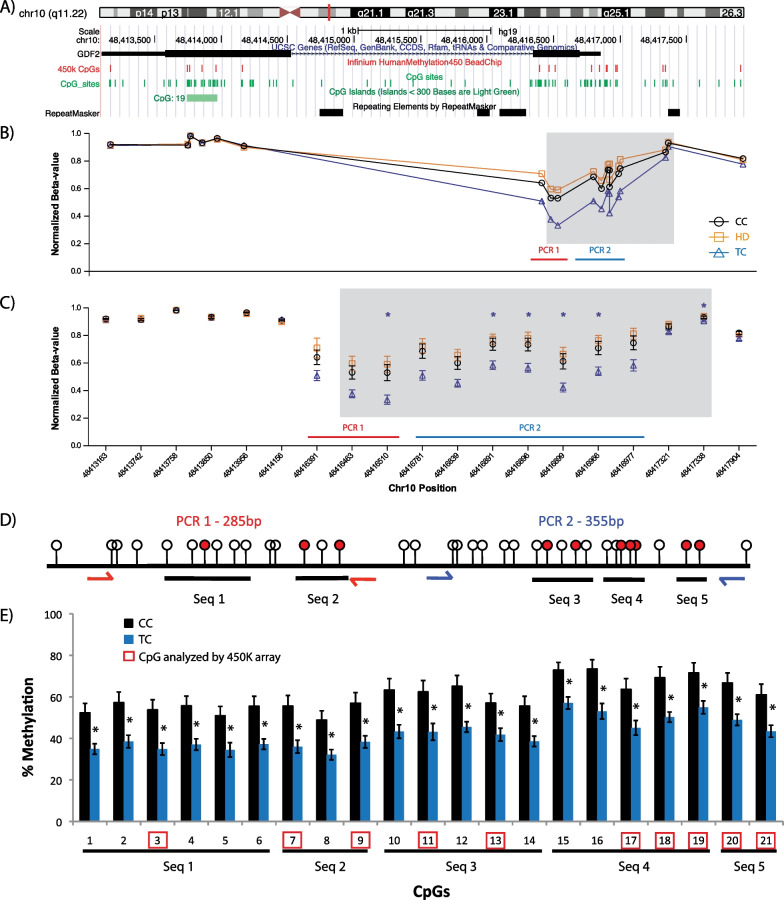
Altered methylation within the *GDF2* promoter is observed exclusively with TC patients. **A** UCSC genome browser view of the *GDF2* gene (grow differentiation factor 2), with tracks showing location of 450 K probes (450 K CpGs) and all CpGs (CpG sites) within the region. Graphs of the average beta-value from each patient groups are depicted, plotting the sites **B** according to their relative position with the genome and **C** separated to indicate SEM and significant CpG sites as discovered following post hoc testing. Highlighted areas in **B** and **C** indicate the DMR discovered following three-group ANOVA, while * indicates significant differences in post hoc Tukey’s testing. **D** A schematic view of CpGs found within promoter region of *GDF2* and pyrosequencing PCR and sequencing primers designed to validate altered methylation. CpG sites analyzed using the 450 K array are indicated. **E** Pyrosequencing results of all CpG sites analyzed between CC and TC patients. Mean ± SEM; *p* < 0.05 *

For the TC group, the largest region that demonstrated differential methylation was found within the promoter–transcription start site (TSS) of the growth differentiation factor 2, *GDF2* (Fig. 5A). For 450 K array probes in *GDF2*, 11 out of 12 CpG sites covered were found to differentiate our cohorts through ANOVA (Fig. 5B and C, shaded box); of those sites, 6 were determined to be altered between TC and CC cohorts following post hoc Tukey’s testing (Fig. 5C). The altered DNA methylation was validated through several bisulfite pyrosequencing assays, which examined a total of 21 CpGs (Fig. 5D). All 21 CpGs examined showed decreased methylation (~ 20%) in TC patients compared to CC (Fig. 5E). Taken together, these results suggest that cancer-specific CpG sites and regions exist for HD and TC patients as compared to CC. While the 450 K array examines only a limited number of CpG sites within a given genomic feature, larger areas of differential methylation may exist that distinguish the different cohorts.

### Increased DNA methylation alterations in a subset of cancer patients following chemotherapy treatment

Next, we examined the effect of chemotherapy on sperm DNA methylation. All CC subjects had at least one time point available following their baseline collection. Conversely, not every cancer patient had samples available for analysis with the 450 K array due to insufficient spermatozoa/DNA post-treatments (HD: 4 patients; TC: 3 patients). Principal component analysis (PCA) using the data from all probes demonstrated that samples from each individual patient had a tendency to group together (Additional file 2: Fig. S5A); however, there are some subjects in which their post-treatments sample was found to be further away from their baseline sample (discussed further below).

**Fig. 6:**
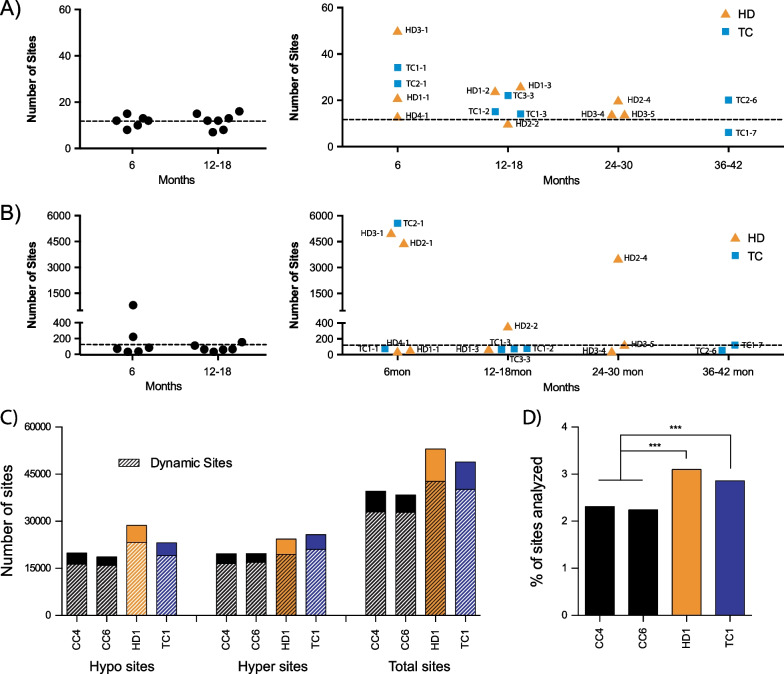
Altered sperm DNA methylation detected months following chemotherapy treatments. **A** RLGS and **B** 450 K array analysis of DNA methylation over time/post-chemotherapy treatment of CC patients (left) and from HD and TC patients (right). The dotted lines drawn within each graph indicate the average number of sites altered in CC patients for each technique used. **C** MCC-Seq (5-methylcytosine capture sequencing) analysis showing the number of CpG sites that demonstrated > 20% methylation difference within individual patients, comparing the 12 and 18 months post-treatment, with the number of dynamic CpG sites indicated. **D** The total number of sites altered is expressed as a percentage of all the sites (1,711,875) analyzed. ***p < 0.0001, chi-squared test with Yate’s correction

Three different genome-wide DNA methylation assays were used to assess the impact of anticancer drug treatment on human sperm. Samples collected from individual patients were compared directly to their own baseline (pre-treatment) sample. For 450 K array data, a cutoff of greater than 0.2 beta-value change (~ 20% methylation) was chosen, similar to the 25% cutoff used for the RLGS approach. Examination of the different time points demonstrated both hyper- and hypomethylation; the total number of sites showing altered methylation for each individual subject using RLGS or the 450 K array is listed in Additional file 2: Fig. S5B-C, respectively. By RLGS, CC individuals showed few loci with altered methylation over time (Fig. 6A, left panel); an average of 12 spots were changed when looking at samples collected at 6, 12 and 18 months compared to baseline. For the cancer survivors, most had an above average number of changes post-chemotherapy (Fig. 6A, right panel); the greatest number of changes, with 50 (~ fourfold increase), was observed for a HD patient, 6 months following treatment (HD3-1). By 450 K array, CC individuals demonstrated on average 145 sites with altered methylation at 6, 12 and 18 months (Fig. 6B, left panel). Compared to this cohort, half of the men undergoing treatment (HD2-1, HD3-1 and TC2-1) demonstrated 20-fold higher alterations in DNA methylation at 6 months post-chemotherapy (Fig. 6B, right panel). While most patients showed similar levels to CC patients at later time points, one HD patient still demonstrated altered sperm DNA methylation at 12 and 24 months (HD2-2 and 4). Samples where an increased number of changes were observed are the same samples that were found to be further away in the PCA from their baseline timepoint (Additional file 2: Fig. S5A). Taken together, our initial results indicate that in some cancer survivors higher than pre-treatment DNA methylation defects in sperm are present, even up to 2 years post-treatment.

We recently developed a human sperm-specific 5-methylcytosine capture sequencing (MCC-Seq) panel that interrogates 3.18 million CpG sites across the genome [31]. The panel targets all sites on Illumina’s MethylationEPIC array (850 K), but also examines CpGs of intermediate levels of methylation (20–80% methylated) found to possess greater variability in human sperm DNA and be susceptible to environmental effects; we refer to the latter as dynamic CpG sites. We hypothesized that the human sperm-specific MCC-Seq panel might be more sensitive than the 450 K array at detecting altered DNA methylation at later time points post-treatment. The capture panel was used to examine two CC and one each of the HD and TC patients. While no baseline time points were available for further analyses, we analyzed samples collected 12 and 18 months post-chemotherapy (time points 2 and 3) and determined the number of differences between these two times, for each subject. With a minimum tenfold coverage, a total of 1,711,875 CpG sites were commonly sequenced between all the samples. Using a similar cutoff as the techniques above, we found that the CC patients had a total of 39,656 and 38,383 CpG sites which demonstrated a minimum 20% difference in methylation; the HD and TC patients both had an elevated number of altered sites, with 53,011 and 48,882 CpGs, respectively (Fig. 6C). In all patients, the number of hypo- and hypermethylation events was similar. Interestingly, a vast majority of the sites found to be different between 12 and 18 months were found in the dynamic sites (hatched bars); on average 82.9% of the changes occurring were at dynamic CpGs. The total number of sites altered from each subject was also expressed as a percentage of all the CpGs analyzed (Fig. 6D); a significantly larger proportion of CpGs was altered in cancer patients as compared to the CC subjects (*p* < 0.0001, chi-squared test with Yate’s correction). The use of the MCC-Seq approach provides further evidence that DNA methylation defects may persist at later time points post-treatment (12–18 months).

### Time-course analysis of post-treatment samples demonstrates altered sperm DNA methylation at 6 months, with some evidence of persistence at later times and common sites affected

**Fig. 7:**
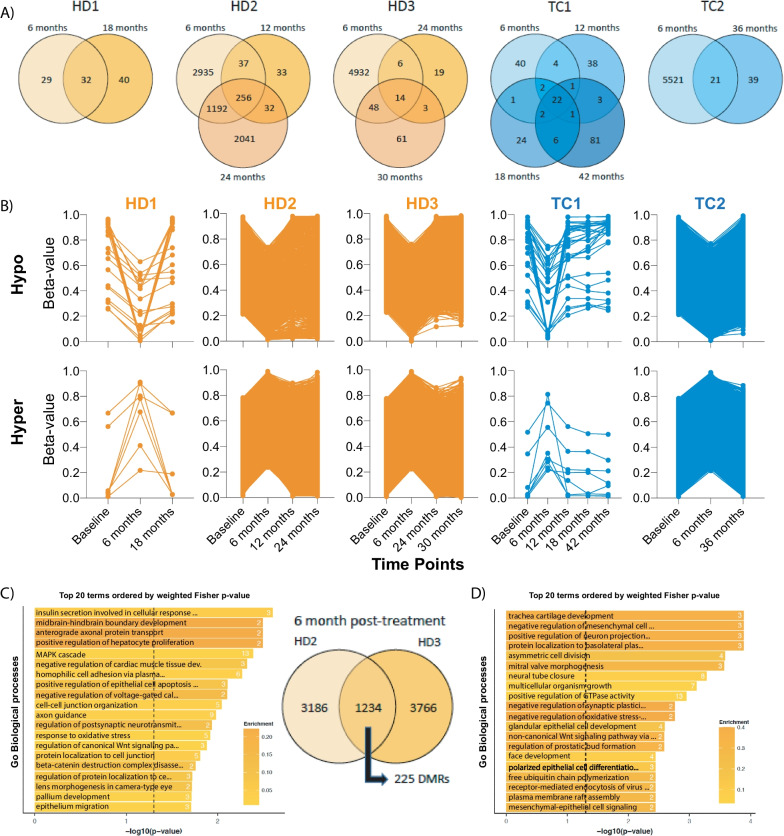
Altered sperm DNA methylation in cancer patients demonstrates mainly acute changes, with some patients showing common alterations and persistent changes over time. **A** Venn diagrams of altered sperm DNA methylation within patients across different collection time points. **B** Acute hypo- and hypermethylation observed at 6 months following chemotherapy treatments. **C** Intersection of DMCs from two HD patients (right) with gene ontology (GO) analysis performed using common DMRs (left). **D** GO analysis of DMCs found to persist throughout all time points from patient HD2. Top 20 most significant GO biological processes, by weighted Fisher *p* values, are plotted with the number in bars representing the number of significant genes annotated to DMRs/DMCs. Enrichment is calculated as a ratio of genes affected and the number of genes found within each GO biological process (see Additional file 1 for all significant GO terms). Dashed vertical line, *p* = 0.05

We next wanted to examine how alterations in sperm DNA methylation behave over time post-treatment, within individual cancer patients, using the 450 k array data. Venn diagrams were created to depict the alterations in the sperm DNA methylome for cancer patients for whom several time points post-treatment were available (Fig. 7A). A preponderance of changes in DNA methylation at the initial 6-month follow-up were specific to this time point. Examining patterns in sperm DNA methylation for these sites across all collection periods, we see that, compared to baseline, hypo- and hypermethylation are observed at 6 months following chemotherapy and that these patterns return to near normal levels at subsequent time points (Fig. 7B).

For patients HD2 and HD3, where a large number of sites were affected at 6 months, we asked whether common sites were affected between patients. A total of 1234 CpGs were altered in both patients, representing ~ 25% of DMCs from each patient; these individual sites were merged into 225 DMRs (Fig. 7C, right). TopGO analysis of these regions revealed a number of significant biological processes, many relating to brain and overall development as well as neurobiology (Fig. 7C, left and Additional file 3). Next, while most changes following treatment appeared to be resolved after the 6-month time point, we asked whether some altered sites persisted over time. For patient HD2, a total of 256 sites were found in common at all the collection times post-chemotherapy (6, 12 and 24 months, Fig. 7A). GO analysis of these sites, found that many biological processes involved in development as well as terms related to the nervous system were significantly enriched (Fig. 7D and Additional file 3). Taken together, while the majority of the defects in sperm DNA methylation patterns immediately following chemotherapy appear to be resolved, common changes due to treatment for HD were identified. These changes and those found to persist up to 2 years post-treatment are related to processes important for development and the nervous system.

### Pre-treatment differential methylation of CpG sites in sperm persists post-chemotherapy treatment

**Fig. 8:**
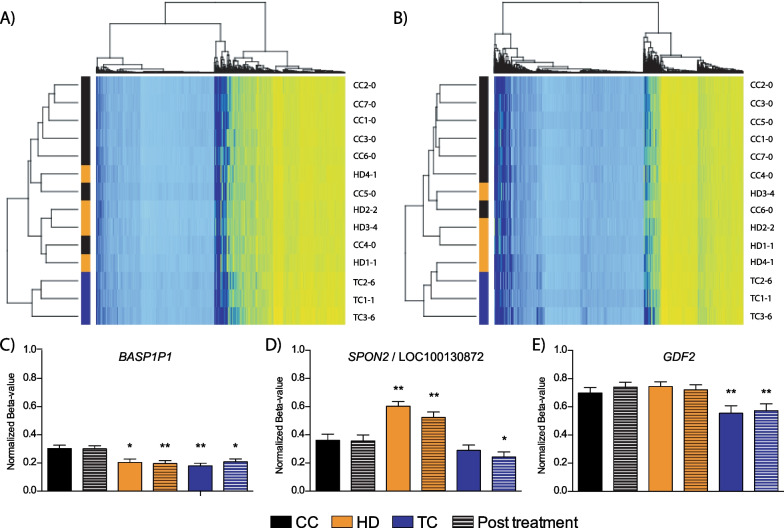
Sperm DNA methylation of samples post-chemotherapy treatment can still distinguish subjects from each other. Hierarchical clustering of cancer patients post-chemotherapy and baseline CC using the previously discovered **A** 11,525 significant ANOVA probes and **B** the top 500 most significant probes. The averaged beta-values of the probes found to differentiate cancer cohorts were used to examine the regions of **C** *SPON2*/LOC100130872, **D** *BASP1P1* and E) *GDF2* in CC, HD and TC at baseline (solid bars) and post-chemotherapy treatments (hatched bars). Mean ± SEM; t test *p < 0.05, **p < 0.01

We postulated that the pre-treatment differences in sperm methylation between the three groups might have resulted from the underlying cancer and would likely disappear post-treatment. To determine if any of the pre-treatment differences between the groups persisted post-treatment, we examined a single sample from each cancer survivor, where the number of altered probes/sites from the 450 K array was similar to that of the CC post-chemotherapy, in an attempt to minimize the effects seen by chemotherapy treatment. In examining the 11,525 significant probes discovered with our baseline (pre-treatment) three-group ANOVA, hierarchical clustering still segregated the TC survivor patients from the HD and CC cohorts post-chemotherapy (Fig. 8A). Using the top 500 significant probes, there was an even better distinction of the three cohorts (Fig. 8B). We next took the 450 K array data from all subjects/patients, regardless of the degree of altered methylation post-treatment, and examined the methylation of DMRs that had shown altered methylation pre-treatment between the different cancer groups. We examined the individual probes discovered through ANOVA, highlighting those specifically altered following post hoc Tukey’s testing, and examined the averaged methylation throughout the region. No significant difference is observed between CC subjects at baseline and at their time points afterwards for the three genes analyzed (Fig. 8C-E and Additional file 2: Fig. S6). In contrast, the *BASP1P1* locus demonstrated significantly lower methylation in all cancer patients at all time points (Fig. 8C and Additional file 2: Fig. S6A). For the region spanning *SPON2*/LOC100130872, HD patients at baseline and post-treatment showed significantly higher levels of methylation (Fig. 8D). Looking at individual CpGs, all sites, including those not significant following post hoc Tukey’s testing, showed increased sperm DNA methylation in HD subjects at all time points (Additional file 2: Fig. S6B). For the *SPON2*/LOC100130872 region, TC patients showed a nonsignificant decrease at baseline (*p* = 0.23), which became significant post-treatment. Similarly, within the promoter-TSS region of *GDF2,* TC subjects demonstrated decreased average methylation post-treatment (Fig. 8E) and all sites across the region possessed lower methylation, as well (Additional file 2: Fig. S6C); this latter result is in line with our pyrosequencing validation which revealed the decreased methylation at many CpG sites across the locus. Taken together, these results indicate that sperm samples collected post-chemotherapy treatment in survivors show persistent differential methylation.

### Sperm DNA methylation within the *GDF2* promoter demonstrates increased variability in TC patients compared to CC in a validation cohort

**Fig. 9:**
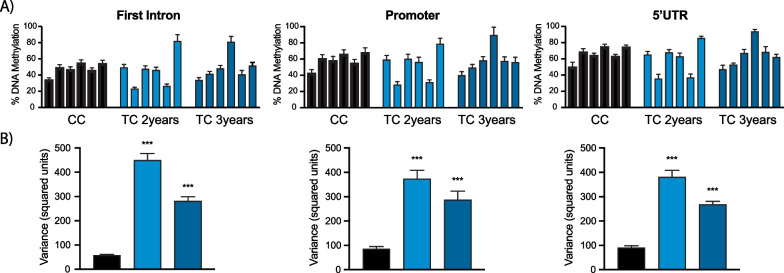
Promoter region of *GDF2* of an external validation cohort demonstrates a large variation in sperm DNA methylation. **A** The averaged methylation of all samples from an external validation cohort is plotted along with the methylation from individual CC and TC patients across the *GFD2* promoter locus; **B** Plot of the average methylation of all samples, from both Canadian and Italian cohorts, in which pyrosequencing results are available. Mean ± SEM; *p < 0.05, Mann–Whitney test

Having demonstrated that, within our study, decreased methylation was observed in TC patients throughout the promoter region of *GDF2* (Fig. 4E) and that this difference is still found in samples post-treatment (Fig. 7E), we wanted to determine whether the same tendencies held true when examining a different cohort (Italian) of subjects. The patient characteristics of these samples are shown in Additional file 1: Table S5. Sperm samples from 6 CC subjects and 12 TC patients, 6 samples each collected at 2 and 3 years post-chemotherapy, were obtained, and bisulfite pyrosequencing was used to assay the same 21 CpG sites within *GDF2*. The average methylation across the locus was similar for the two groups (55.71% and 54.17% for CC and TC, respectively). However, there was more variability in *GDF2* methylation among the TC patients, particularly at 2 years post-treatment, where 2 of the 6 patients showed low levels of methylation across the locus (Fig. 9A). Variability in *GDF2* methylation decreased but remained significant at 3 years post-treatment (Fig. 9B).

## Discussion

Hodgkin disease and testicular cancer are two forms of cancers that are commonly diagnosed in men of young reproductive age. While treatments result in high survival rates, questions about fertility and the possibility of fathering children post-treatment are of particular concern. Are there negative impacts due to the disease state itself and/or caused by the chemotherapy treatments that can affect the sperm? Can these have implications for the next generation? This study adds to previous work, including reports from our group, demonstrating an increased incidence of aneuploidy, DNA damage and altered chromatin quality in human sperm up to 2 years following combination chemotherapy treatments for HD and TC [7–10]. Our findings here reveal distinct DNA methylation patterning in sperm prior to the onset of treatment that are able to distinguish HD, TC and CC subject groups from one another; interestingly, these profiles between the different groups remained post-treatment. As well, by comparing baseline and post-chemotherapy samples, while most initial defects appear to be resolved at later time points, altered sperm DNA methylation was detected in patients up to 18–24 months post-treatment, indicating that treatment-induced epigenetic abnormalities, like genetic damage, persist long after patients become disease free.

Imprinted genes acquire sperm-specific DNA methylation patterns during male germ cell development and play crucial roles in normal development; disturbances in genomic imprinting can lead to a number of disorders [32]. We analyzed the methylation of several imprinted genes represented on the 450 K arrays and found normal profiles in the sperm of men diagnosed with HD or TC, prior to, and following, combination chemotherapy treatments. Our pre-treatment results are in keeping with those of a recent study examining the DNA methylation status of imprinted genes in cryopreserved sperm samples from normospermic before treatment seminoma patients and control subjects, where no alterations in imprinted gene methylation were found [33]. The absence of imprinted gene DNA methylation abnormalities in testicular cancer patients prior to treatment in two different studies is reassuring. We are not aware of studies that have compared imprinted gene methylation in patients before and after treatment. However, we previously examined effects of human testicular cancer treatment regimens (BEP) on sperm DNA methylation in a rat model [17]. Similar to our human studies, imprinted gene methylation was not affected by the BEP treatment. In a post-treatment genome-wide study of sperm DNA methylation in adult patients 10 years after pubertal exposure to chemotherapy for osteosarcoma, no imprinting gene methylation abnormalities were reported [6].

The study presented here is novel, in that we were able to examine the human sperm DNA methylome in samples acquired prior to the initiation of treatment and follow the same patients at several time points post-chemotherapy. Our results with the 450 K arrays demonstrated that, prior to treatment, sperm DNA methylation signatures are present in men and are able to distinguish HD and, to a greater extent, TC patients from CC subjects. Interestingly, these group-specific DNA methylation patterns persisted in sperm following chemotherapy, up to the 3.5-year the period studied. Pre-treatment signatures were not evident using the lower resolution RLGS method. RLGS examines DNA methylation at a relatively small number of loci compared to the 450 K arrays and can only reproducibly detect larger changes in DNA methylation, greater than 25% in magnitude. Similar to our findings, sperm aneuploidy has also been detected in pre-treatment samples from testicular cancer and Hodgkin disease patients [7, 34]. Explanations for the presence of pre-treatment signatures include the impact of a cancer environment on germline stem cells, underlying genetic susceptibility, and an altered somatic environment. Recent studies examining the incidence of birth defects in the children of men with cancer, support the potential clinical relevance of the presence of pre-treatment genetic and epigenetic alterations in the spermatozoa of cancer patients [13]. While studying sperm aneuploidy is labor-intensive and requires specialized expertise, genome-wide DNA methylation assays are more widely accessible and may facilitate the screening of pre-treatment spermatozoa for prospective studies.

Our study suggests that pre-treatment DNA methylation differences between groups can persist post-treatment. In support of the finding in our prospective cohort, validation of a locus found to be altered in TC patients also showed greater variation in an external cohort, where samples were collected 2–3 years following chemotherapy. The most likely explanation would be persistence of abnormal germline stem cells or spermatogonial stem cells (SSCs) in the testis post-treatment. SSCs that had been quiescent may not have been eliminated by the chemotherapy. That SSCs with abnormal DNA methylation patterns can persist in the testis for many years is supported by a study of osteosarcoma patients treated as adolescents and examined as adults [6]. Although pre-treatment samples were not available for the latter study, altered DNA methylation patterns were found in spermatozoa 10 years after pubertal exposure to chemotherapy.

The pre-treatment aberrant epigenetic marks found in the sperm of HD and TC patients may identify a preexisting signature that could be used to screen patients. Indeed, both diseases have etiologies associated with altered epigenetic patterning. The distinctive cells used for the diagnosis of HD, the Hodgkin/Reed–Sternberg cells, have been shown to have lost or decreased many of the B cell-specific gene expression profiles [35, 36]. By altering DNA methylation and/or histone acetylation patterning, studies in cell lines have been able to alter the expression patterns of B cells to resemble more closely those characteristics of HD, and vice versa [37–39]. Testicular cancer, hypospadias, cryptorchidism and poor semen quality, are included in the clinical entity, testicular dysgenesis syndrome, a disorder postulated to be due to environmental effects that disrupt embryonic and fetal germ cell development [40, 41]. Indeed, the precursor of testicular germ cell tumors, intratubular germ cell neoplasia (ITGCN) cells, has been theorized to originate from fetal gonocytes, through morphological and gene expression profiling [42, 43]. Epigenetic marks of the ITGCN have also been demonstrated to be similar to those of fetal germ cells [44, 45], allowing for increased proliferative activity. Interestingly, biological processes relating to the immune system/immune response and sexual/male reproduction were observed in the differentially methylated regions from the baseline analysis of the HD and TC cohorts, respectively, alluding to the origins of these diseases. While the altered epigenetic signatures from our patient cohorts were discovered in sperm, it remains to be determined whether more accessible biological samples, such as blood or saliva, would also carry perturbed epigenetic patterning.

From cancer studies, methylation of loci identified in both HD and TC patient sperm are known to influence gene expression. The largest differentially methylated region found in HD patients was within the *SPON2* locus, showing increased methylation in HD versus CC individuals. Altered methylation within the promoter of this extracellular matrix protein has been observed both in prostate cancer and in cancer-associated myofibroblast cell lines from gastric and esophageal adenocarcinomas compared to normal tissues [46, 47]. Decreased methylation within the promoter led to a corresponding increase in *SPON2* expression within the cell lines, which was also detected in patients with the poorer prognosis and survival of gastric/colorectal carcinoma patients [48, 49]. Specific to testicular cancer, decreased methylation in sperm was observed within the promoter of *GDF2* in our patient cohort, a result also seen in a subset of TC patients 2–3 years post-treatment in the validation cohort. This locus encodes the GDF2 protein, also known as bone morphogenic protein 9 (BMP9), a member of the TGFβ superfamily, which has roles in cancer progression [50]. Specifically, GDF2 activation of SMAD1/5 signaling has been found to enhance cell death in ovarian and breast cancer cell lines; treatment of these cells with 5-azacytidine, a potent demethylating agent, demonstrated decreased methylation of the *GDF2* locus, increased signaling and higher sensitivity to cell death [51]. This same study also observed higher *GDF2* promoter methylation in tumors of ovarian cancer patients compared to normal individuals. Similarly, loss of *GDF2/BMP9*, in a mouse model of breast cancer, was associated with increased tumor growth and metastasis, indicating a protective role of this gene [52]. From the cancer studies, the increased methylation of *SPON2* in the sperm of HD patients would result in decreased expression, while the decreased methylation of *GDF2* in the sperm of the TC patients would result in increased expression. We hypothesize that such altered expression patterns, perhaps involving other genes identified in the HD and TC cohorts, may provide a growth or differentiation advantage for germ cells.

Immediately following chemotherapy treatments, many patients in the HD and TC cohorts showed an elevated number of sites with altered sperm DNA methylation compared to the CC groups. Indeed, a significant increase in DNA damage, as measured by tail extent moment (Comet assay), was detected in a similar group of both HD and TC patients 6 months after treatments [9]. We also observed that many of the initial defects observed were resolved and the number of altered sites diminished with time, similar to the treatment-induced damage to sperm chromatin structure being differentially repaired over time in cancer survivors. However, one patient demonstrated increased alterations in the sperm methylome in the following 2 years; similar significant sperm DNA damage and low DNA compaction have been observed up to 24 months post-treatment [10]. It is also intriguing that developmental and nervous system pathways were enriched in examining our post-treatment effects for GO biological process terms. Taken together, alterations in sperm DNA methylation, altered sperm chromatin structure and DNA damage can be observed in HD and TC patients prior to and up to several years following chemotherapy regimens. This damage to the genetic/epigenetic cargo of spermatozoa may help to explain the observed increased risk of congenital abnormalities in children fathered by men with a history of cancer, whether the offspring were conceived prior to or years following treatments [11, 12].

One limitation of our study is the small number of patients available in each cohort, as well as the reduced numbers of samples available following treatments. While these are common forms of cancers in men of reproductive age, the incidence is still low, with 2.8 and 6.8 per 100,000 in the United States for HD and TC, respectively [53, 54]. As well, the impact of treatments on spermatogenesis is evident with decreased sperm counts immediately following chemotherapy, which only return to pre-treatment levels after 24 months [55]. Despite the low patient numbers in our different cohorts, we were able to detect differential methylation signatures in sperm. As mentioned previously, a validation in other cohorts of HD and TC patients of these signatures would be beneficial. Another limitation is that we were only able to examine a subset of the ~ 32.8 million possible CpG methylation sites found in the human genome [56]. The majority of the results presented here are from the Illumina 450 K array, examining approximately 450,000 CpG sites, with these mainly covering CpG islands and over gene regions. Our early study examining sperm DNA methylation using the 450 K array, showed that the majority of CpGs interrogated (86% of probes) were hypo- or hypermethylated (< 20% or > 80%, respectively) with methylation levels conserved between individuals; only a small proportion of probes (0.3%) were variable between individuals and demonstrated intermediate (20–80%) methylation [57]. We reported similar finding (i.e., a minority of variable CpG sites) in a more recent study using the 450 K array and examining the effects of low dose folic acid supplementation on sperm DNA methylation [58]. Our use of the human sperm-specific MCC-Seq that examines 3.18 million sites, did demonstrate a greater number of alterations in patients who underwent chemotherapy treatments; the vast majority of the altered DNA methylation was found in dynamic CpGs, sites of intermediate methylation and not well covered on the different Illumina arrays. Indeed, the use of our sperm-specific panel for the analysis of human DNA methylation differences due to a genetic polymorphism, environmental exposures, as well as the process of aging, has demonstrated that differential methylation is mainly found in these novel dynamic CpG sites targeted [31, 59]. Future studies using our human sperm targeted panel, or with whole-genome bisulfite sequencing, may be useful to examine in greater detail the effect of cancer as well as their treatments on the sperm methylome.

## Conclusion

This is the first study that demonstrates distinct sperm DNA methylation signatures in cancer patient cohorts: differences in the sperm DNA methylome between CC, HD and TC patients were observed prior to the initiation of treatment and these signatures persisted post-chemotherapy, up to the 3.5-year time period studied. Following treatments, we observed higher levels of methylation defects, which decreased over time. However, similar to observed DNA damage, in some patients, these defects persisted up to 2 years following treatment. Using a targeted human sperm capture sequencing technique, differences in sperm DNA methylation were observed in novel dynamic CpG sites, which warrants further investigation. Our findings emphasize the importance of collecting pre-treatment samples in future studies and may help explain increases in birth defects reported in cancer patients both before and after their treatment.

## Methods

### Semen sample collection from cancer patients and community controls

Semen samples were obtained from a cohort of subjects with non-seminoma testicular cancer (TC, stages II–III, *n* = 7) or Hodgkin lymphoma (HD, stage II–IV, *n* = 7) who underwent combination chemotherapy and no radiation therapy treatments; TC patients were treated with 2–4 cycles of BEP chemotherapy (bleomycin, etoposide, cisplatin) and HD patients underwent 4–8 cycles of ABVD treatments (doxorubicin, bleomycin, vinblastine, dacarbazine). Collection time points from these individuals were at baseline (*t* = 0), prior to initiation of chemotherapy, and at 6 months intervals (*t* = 1, 2, 3, etc.), up to 3.5 years, after termination of treatments. Due to dropout and/or azoospermia, samples could not be obtained throughout the post-chemotherapy period for all patients. A cohort of healthy untreated community control (CC, *n* = 8) volunteers was also recruited. The CC subjects had no significant past medical history or history of infertility. All CC, HD and TC subjects were non-smokers and were part of a larger cohort of subjects, where studies examining sperm quality and damage over time and following chemotherapy treatments were previously conducted [8, 9]. The baseline samples from the CC cohort were included as a post mandatory folic acid fortification group in a previous study of ours [58]. Sperm from men in all groups were collected during the same time period; the men would have been exposed to folic acid fortification in their diets, due to the mandatory addition of this vitamin to white flour and enriched grain products in 1998; no additional folic acid supplementation was given to any of the cohorts. Semen samples were collected by masturbation, with a minimum of 3 days abstinence and allowed to liquefy at 37 °C for 30 min. Samples were then aliquoted and stored at −80 °C in the absence of cryo-protectant. Samples from an Italian validation cohort, were also collected in a similar fashion. Here, TC patient survivors who had undergone 1–4 cycles of BEP chemotherapy were recruited and samples collected 2 or 3 years after termination of treatments (*n* = 6–3 seminoma and 3 non-seminoma, per time point); six control samples from this cohort were also examined. The study was approved by the different institutional ethics review boards and informed consent was obtained from all subjects (see Declarations).

### Sperm DNA extraction

Semen samples were thawed on ice and centrifuged at 4000 g for 5 min in order to obtain sperm pellets. Pellets were resuspended in lysis buffer (10 mM Tris•HCl, pH 8; 150 mM EDTA, pH 8; 100 mM DTT) and high molecular weight DNA isolated by Proteinase K digestion and phenol–chloroform extraction, as described previously [60]. Precipitated DNA was allowed to dissolve in TE buffer (10 mM Tris•HCl, pH 8; 1 mM EDTA) for 1 week before proceeding with DNA methylation analyses.

### Global assessment of methylation by RLGS

Several techniques were used for the assessment of DNA methylation. Initial analysis relied on restriction landmark genomic scanning, RLGS [60]. Briefly, DNA samples (CC, *n* = 8; HD, *n* = 7; TC, *n* = 6) were digested with the methylation sensitive restriction enzyme, NotI. Following this, radioactive end-labeling of overhangs was performed, proceeded by further digestion of samples with EcoRV. DNA fragments were electrophoresed on a first dimension agarose gel and in situ digestion with a third restriction enzyme, Hinf1, was carried out. Samples were finally run on a second dimension acrylamide gel, resulting in the final RLGS profile. Profiles were imaged on phosphorimaging plates and film. Films were visually assessed and a scoring system previously described was used for analysis [61].

### Methylation profiling with HumanMethylation450 BeadChip

Illumina’s Infinium HumanMethylation 450 BeadChip (450 K) array was used in order to assess the methylation at over 480,000 CpGs dinucleotides of CC (*n* = 8), HD (*n* = 7) and TC (*n* = 7) subjects. The assay was conducted by Genome Quebec at the McGill University Innovation Centre using a protocol previously described [62]. Briefly, ~ 500 ng of DNA was bisulfite converted using an EZ DNA methylation Kit (Zymo Research), followed by whole-genome amplification, enzymatic fragmentation and hybridization to the BeadChip. Arrays were imaged on the Illumina iScan, resulting in raw data files in IDAT format. The annotation of the 450 K array to determine the genomic location of probes was determined with IlluminaHumanMethylation450kanno.ilmn12.hg19 (version 0.6.0) [63]. Preprocessing of the raw data files (IDAT), quality control (QC) assessment and normalization were performed using the R package minfi [64], with normalization based on the study by Fortin et al. [65]. The QC demonstrated that a single sample from a CC patient (CC7-2) clustered separately from other samples and failed due to lower median intensities (Additional file 2: Fig. S7A, red arrow). PCA of raw and normalized data was also carried out using the R package shiny methyl (version 1.22.0) [66]. This analysis indicated that the same sample failing QC was an outlier of PC2 (Additional file 2: Fig. S7B, red arrow); as well, three other samples (one CC and two TC; CC8-0, TC7-0 and TC7-1; blue arrows) appeared to be outliers within PC1. We examined whether these samples were possibly contaminated, by examining the imprinted gene methylation. The three samples were identified as outliers through PCA analysis, which demonstrated deviation in methylation across all germline ICRs analyzed to a similar extent; this would indicate somatic cell contamination (Additional file 1: Table S1). Hierarchical clustering of the averaged methylation from all ICRs of imprinted genes demonstrated that the failed QC sample as well as the outliers clustered clearly apart (Additional file 2: Fig. S7C); therefore, all these (along with any other post-treatment samples) were removed from further analysis, leaving an *n* = 7 for the CC and HD cohorts and an *n* = 6 for the TC cohort.

### Customized 5-Methyl-C capture sequencing (MCC-Seq) of DNA with human sperm-specific panel

Our recently developed human sperm-specific capture panel [31] was used to assess over 3 million CpG sites within the genome of 8 samples: two CC and one each HD and TC at times 2 and 3 following chemotherapy treatment. Using 1 µg of DNA from each sample, whole-genome bisulfite sequencing (WGBS) library preparation and targeting bisulfite sequencing were performed as described previously [67, 68]. Briefly, DNA was sonicated to obtain 300–400 bp fragments, proceeded by DNA-end repair, 3’-end adenylation, adaptor ligation and clean up, according to the KAPA High Throughput Library Preparation kit protocol (Roche/KAPA Biosystems). Samples were then bisulfite converted using the EpiTect Fast DNA bisulfite kit (Qiagen) followed by 9–12 cycles of PCR amplification. The final WGBS libraries were purified using Agencourt AMPure Beads (Beckman Coulter) and were then enriched using our human sperm-specific panel, using the SeqCap Epi Enrichment System protocol developed and optimized by Roche NimbleGen. Equal amounts of the multiplexed WGBS libraries (84 ng each) were combined and hybridized to the human sperm-specific capture panel at 47 °C for 72 h. The captured libraries were washed, recovered and PCR-amplified before final purification was conducted. The final capture libraries were sequenced in a single lane of the Illumina HiSeq4000 system using 100-bp paired end sequencing. Raw reads from the sequencers were processed using pipelines developed at the McGill University and Génome Québec Innovation Centre (MUGQIC), as part of the GenAP project (https://bitbucket.org/mugqic/genpipes). Common CpG sites from all samples, with > 10 × coverage, were retained.

### Locus-specific analysis of DNA methylation by bisulfite pyrosequencing

Bisulfite pyrosequencing was used to validate differential methylation at the promoter region of *GDF2*. Here, baseline sperm DNA samples prior to any treatments were analyzed from CC (*n* = 8) and TC (*n* = 5) cohorts of our main study, as well the Italian validation cohort. For pyrosequencing assays, sperm DNA (200–500 ng) underwent bisulfite conversion with the EpiTect Bisulfite Kit (Qiagen) according to the manufacturer’s protocol. Primers were designed (PyroMark Assay Design 2.0, Qiagen) to overlap the areas where differential methylation was observed following 450 K array analysis; two sets of PCR primers, using a total of five sequencing primers, were designed in order to analyze a total of 21 CpG sites (Additional file 1: Table S6). Bisulfite PCR was conducted, and pyrosequencing was performed as previously described [69] using the PyroMark Q24 kit (Qiagen) as per the manufacturer’s protocol.

### Statistical analyses

The R environment and language (version 3.5.2) were used to perform statistical computing and graphing of the of 450 K array data. Specifically, graphical heatmaps and hierarchical clustering, as well as ANOVA and post hoc Tukey’s tests, were conducted using RStudio (version 1.2.1335). Significant probes within 1000 bp were merged using the merge function of BEDtools (version 2.29.0) to determine DMRs. Other graphs were generated, and statistical analyses were performed using GraphPad Prism Version 6.01. Unpaired t tests were performed to compare the mean sperm DNA methylation of each CpG analyzed by bisulfite pyrosequencing, as well as the averaged normalized beta-value across the different DMRs. GO term enrichment was performed using the R package topGO (version. 2.42.0). Chi-squared with Yates’ correction was applied to compare the frequency of CpG sites having > 20% methylation changed, as measured by MCC-Seq, in cancer patients compared to the individual CC subjects. Unless otherwise specified, statistical significance was set at *p* < 0.05 for the different analyses.

## Supplementary Information

**Additional file 1**. Supplementary Tables S1–S6.

**Additional file 2**. Supplementaion Figures S1–S7.

**Additional file 3**. Complete list of significant GO terms.

## Data Availability

Illumina HumanMethylation 450 K BeadChip data (IDAT files) and MCC-Seq data have been submitted to Gene Expression Omnibus (GEO) under the accession number GSE211122.

## Abbreviations

TC: Testicular cancer
HD: Hodgkin disease
CC: Community control
450 k array: Illumina HumanMethylation 450 K BeadChip
PCA: Principal component analysis
ICR: Imprinting control region
RLGS: Restriction landmark genome scanning
DMR: Differentially methylated region
TSS: Transcriptional start site
MCC-seq: 5-Methylcytosine capture sequencing
BEP treatments: Bleomycin, etoposide, cisplatin treatments
ABVD treatments: Doxorubicin, bleomycin, vinblastine, dacarbazine treatments
