# Supplementary material for: Sperm DNA methylome abnormalities occur both pre- and post-treatment in men with Hodgkin disease and testicular cancer
Source: Clin Epigenetics. 2023 Jan 7;15:5. doi: 10.1186/s13148-022-01417-1 (PMC9826600; doi:10.1186/s13148-022-01417-1)
Supplement: Supplementary file 2 — Additional file 2. Supplementaion Figures S1–S7. [file 13148_2022_1417_MOESM2_ESM.pdf]

Sperm DNA methylome abnormalities occur both pre- and post-treatment in men with Hodgkin disease and testicular cancer

Authors: Donovan Chan, Kathleen Oros Klein, Antoni Riera-Escamilla, Csilla Krausz, Cristian O'Flaherty, Peter Chan, Bernard Robaire, Jacquetta M. Trasler*

*Corresponding author

<u>**Additional File 2 containing Supplemental Figures S1-S7**</u>

Supp. Figure S1. Heatmap and hierarchal clustering of original and randomized probes.

Supp. Figure S2. GO analysis of differentially methylated regions.

Supp. Figure S3. RLGS analysis of baseline samples from CC, HD and TC subjects.

Supp. Figure S4. Altered sperm DNA methylation between chemotherapy patients compared with CC.

Supp. Figure S5. Analysis of DNA methylation changes following chemotherapy treatments.

Supp. Figure S6. Individual probe beta-value/methylation of 450K array data pre- and post-chemotherapy treatments.

Supp. Figure S7. Removal of samples failing probe quality control and/or have contamination.

FIGURE S1

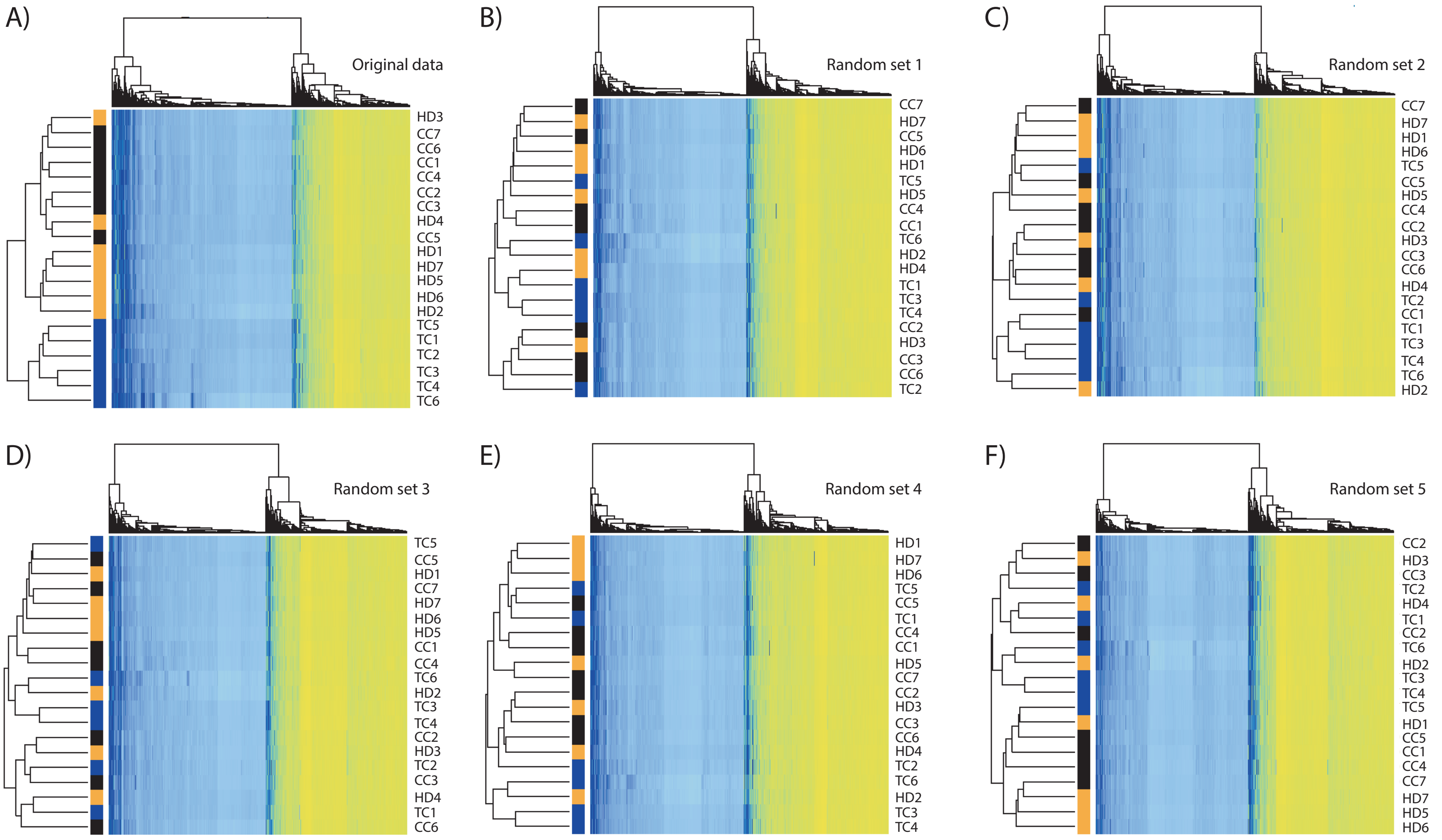

Supp. Figure S1. Heatmap and hierarchal clustering of original and randomized probes
Hierarchal clustering of A) the original 11,525 probes discovered by 3-group ANOVA demonstrates good clustering of TC, HD and CC cohort while B-F) clustering of the same number of randomly selected probes shows no similar grouping of the different cohorts.

FIGURE S2

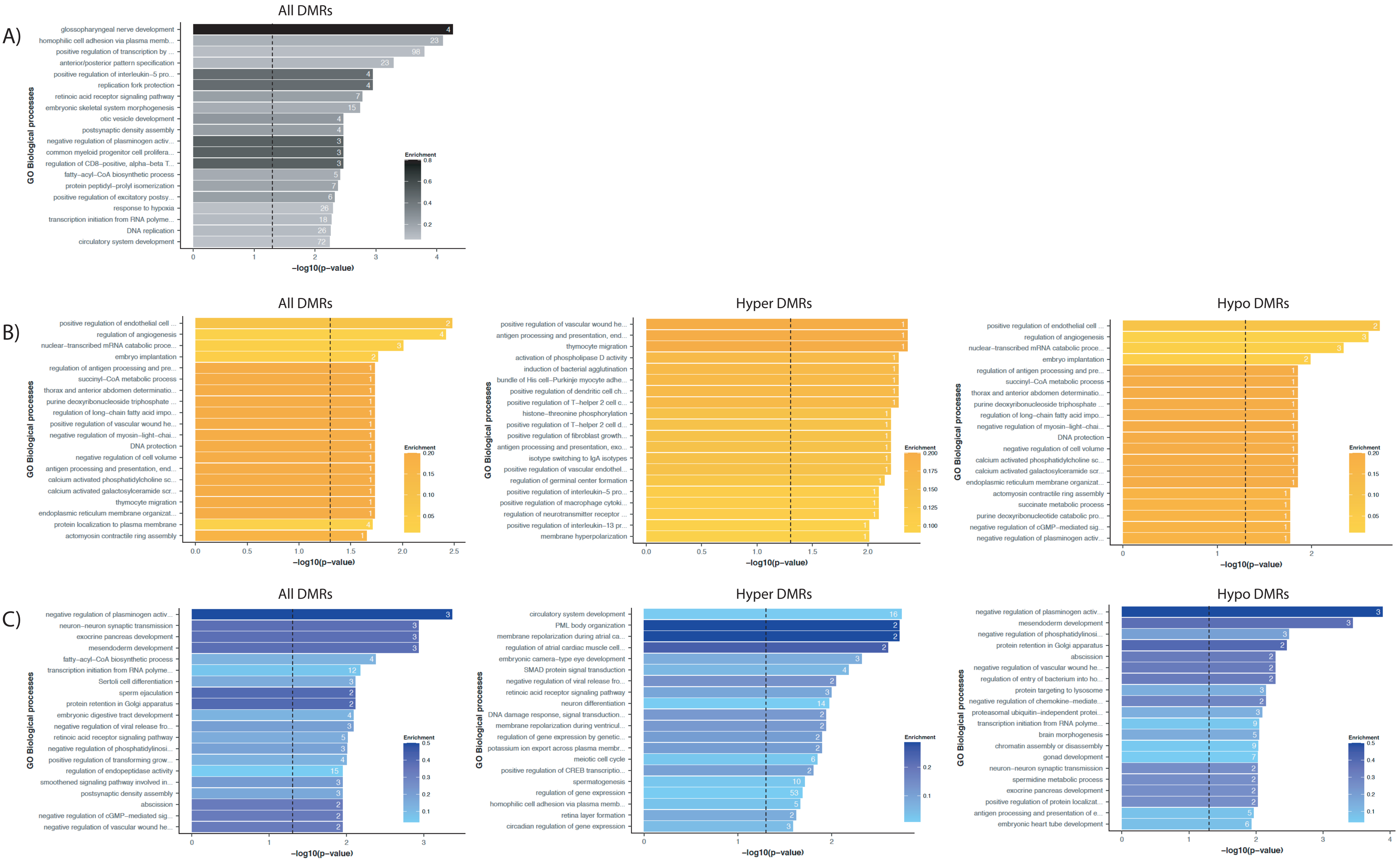

Supp. Figure S2. GO analysis of differentially methylated regions.
TopGO pathway analysis was used to determine gene ontology (GO) Biological process terms enriched and the top 20 significant terms from differentially methylated regions (DMRs) discovered from baseline samples following A) 3-group ANOVA (related to Figure 2) or post-hoc B) HD vs. CC or C) TC vs. CC analyses (related to Figure 4). GO was determined using all DMRs (left) or using hyper- (centre) or hypo-methylated (right) DMRs separately. Verical dashed lines represent a weighted Fisher p-value of 0.05. The numbers indicated within each bar represent the number of significant DMRs genes annotated within each GO term. Enrichment is calculated as a ratio DMRs genes found and the total number of genes within each GO term (see supplement File 1 for all significant and full names of GO terms).

FIGURE S3

A)

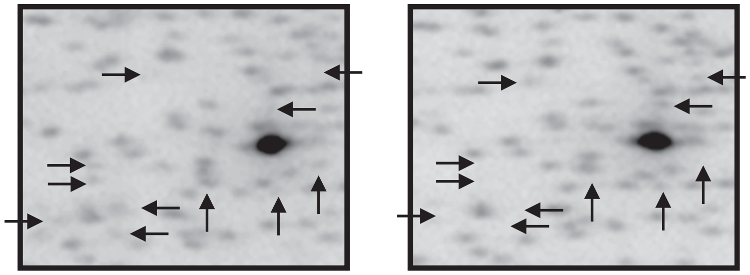

B)

Patient	Community Control (CC)	Hodgkins Disease (HD)	Testicular Cancer (TC)
1	81	83	97
2	78	66	90
3	94	75	111
4	94	98	98
5	76	102	102
6	103	102	90
7	Reference	NA	-

Supp. Figure S3. RLGS analysis of baseline samples from CC, HD and TC subjects
A) Comparison of RLGS baseline gels indicating differences observed between subjects (50-100% methylation). B) Number of observed differences when compared to a common CC RLGS profile. No differentially methylated locus/spot was found solely in the cancer groups compared to the CC cohort (i.e. do cancer spefic loci determined by RLGS.)

FIGURE S4

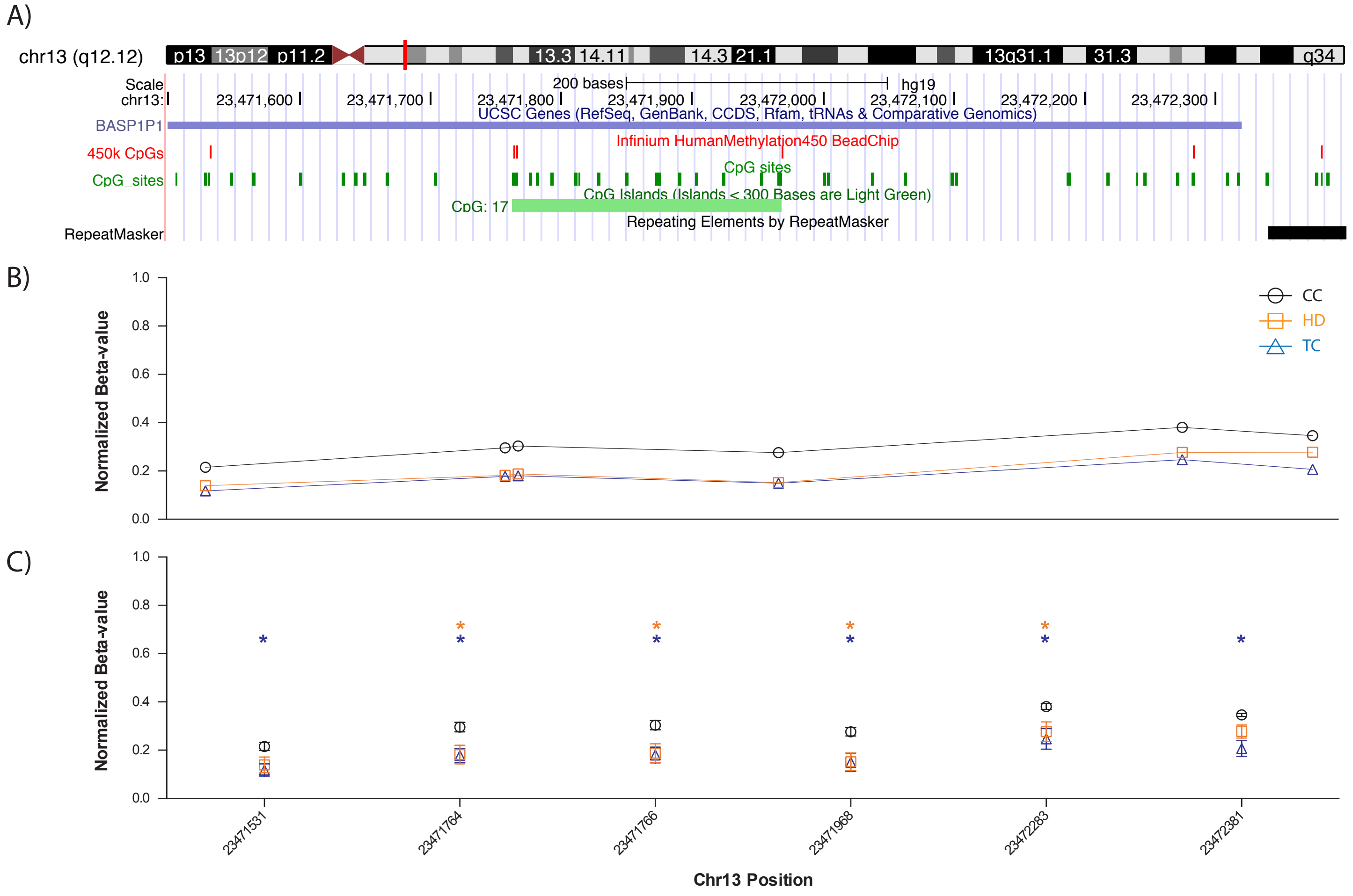

Supp. Figure S4. Altered sperm DNA methylation between chemotherapy patients compared with CC.
A) UCSC genome browser view of the non-coding RNA of *BASP1P1* (brain abundant, membrane attached signal protein pseudogene 1), with tracks showing location of 450k probes (450k CpGs) and all CpGs (CpG sites) within the region. Graphs of the average Beta-value from each patient groups is depicted between the 3 different patient groups, plotting the sites B) according to their relative position with the region and C) separated to indi cate SEM and significant CpG sites. Mean +/- SEM, $p < 0.05$ *

FIGURE S5

A)

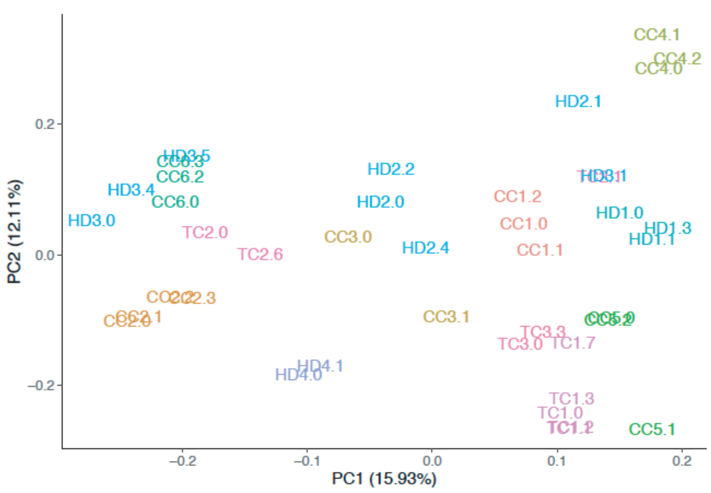

B)

Sample	Total (Hyper/Hypo)						
	1	2	3	4	5	6	7
CC1	15 (14/1)	13 (13/0)	-	-	-	-	-
CC2	13 (11/2)	8 (5/3)	15 (6/9)	-	-	-	-
CC3	12 (12/0)	16 (15/1)	-	-	-	-	-
CC4	10 (4/6)	-	12 (8/4)	-	-	-	-
CC5	12 (12/0)	12 (9/3)	-	-	-	-	-
CC6	8 (6/2)	7 (6/1)					
HD1	21 (14/7)	24 (19/5)	26 (20/6)	-	-	-	-
HD2	-	10 (3/7)	-	20 (6/14)	-	-	-
HD3	50 (17/33)	-	-	14 3/11)	14 (1/13)	-	-
HD4	13 (11/2)	-	-	-	-	-	-
TC1	34 (32/2)	15 (3/12)	14 (6/8)	-	-	-	20 (16/4)
TC2	27 (18/9)	-	-	-	-	6 (6/0)	-
TC3	-	-	22 (7/15)	-	-	-	-

C)

Sample	Total (Hyper/Hypo)						
	1	2	3	4	5	6	7
CC1	71 (33/38)	59 (31/28)	-	-	-	-	-
CC2	84 (47/37)	111 (80/31)	154 (116/38)	-	-	-	-
CC3	221 132/86)	-	-	-	-	-	-
CC4	32 (19/13)	66 (26/40)	-				
CC5	812 (59/753)	59 (31/28)	-	-	-	-	-
CC6	37 (25/12)	32 (20/12)	-	-	-	-	-
HD1	61 (24/37)	-	72 (28/44)	-	-	-	-
HD2	4420 (2983/1437)	358 (46/212)	-	3521 (1215/2306)	-	-	-
HD3	5000 (2541/2459)	-	-	42 (22/20)	126 (95/31)	-	-
HD4	43 (26/17)	-	-	-	-	-	-
TC1	73 (31/42)	75 (34/41)	62 (31/31)	-	-	-	117 (97/20)
TC2	5542 (3310/2232)	-	-	-	-	60 (22/38)	-
TC3	-	-	70 (16/54)	-	-	-	-

Supp. Figure S5. Analysis of DNA methylation changes following chemotherapy treatments.
A) Principal compoent analysis (PCA) of all samples from the different times points (i.e. 0, 1, 2, 3, 4, 5, 6 and 7, representing baseline, 6, 12, 18, 24, 30, 36, and 42 months post-treatment), colored by individual patients. Tables indicating the total number of changes (hyper/hypo) observed through B) RLGS and C) 450K array comparing different time points to their respective baseline samples.

FIGURE S6

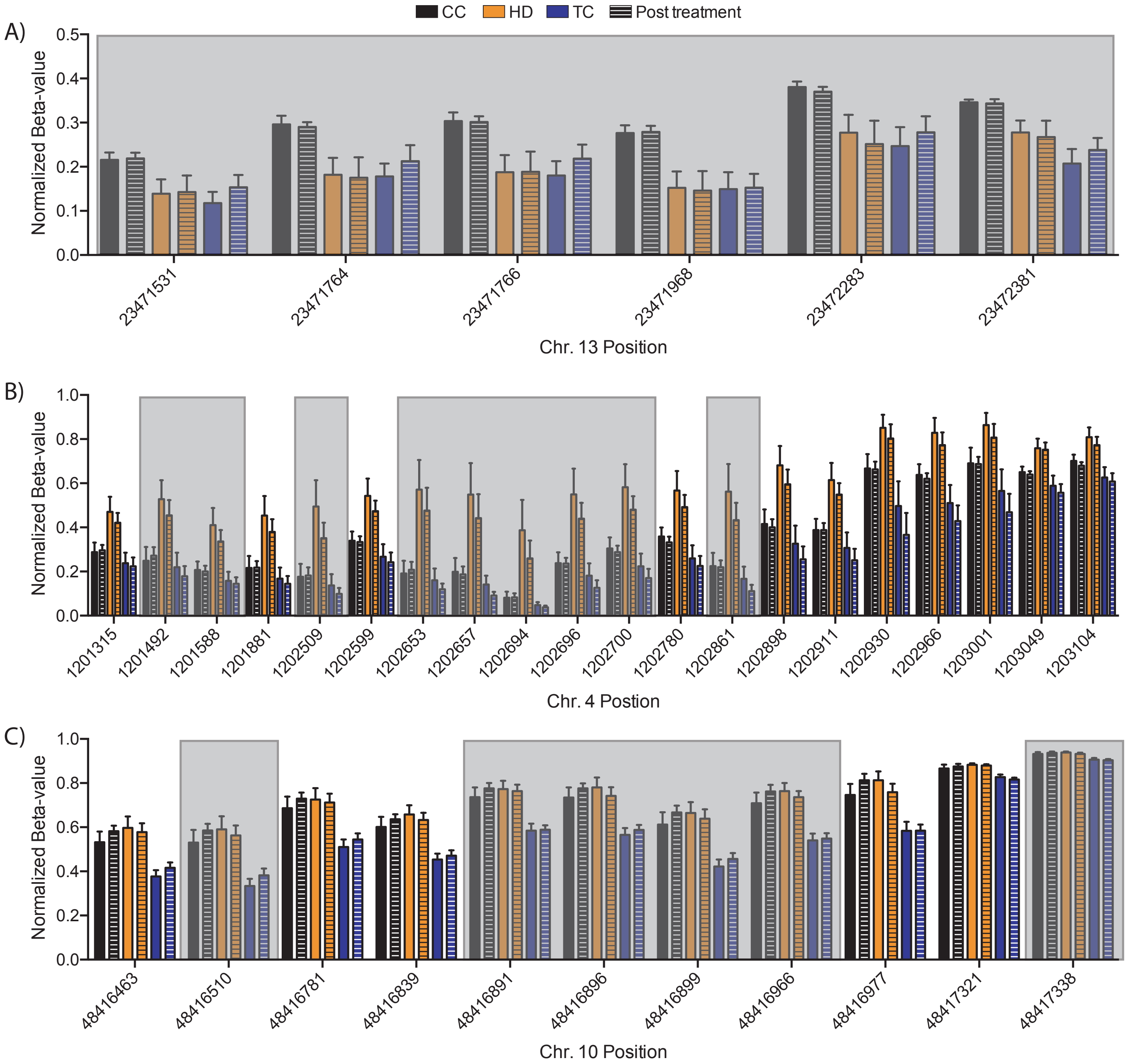

Supp. Figure S6. Individual probe beta-value/methylation of 450K array data pre- and post-chemotherapy treatments.

The average beta-value/methylation for individual probes was determined within the regions of A) *BASP1P1*, B) *SPON2*/LOC100130872 and C) *GDF2* in the three different cohorts at baseline (pre-treatment; solid bars) and a time points post-treatment (hatched bars). Boxed areas represent sites discovered to be significant by post-hoc Tukey's testing. Mean +/- SEM.

FIGURE S7

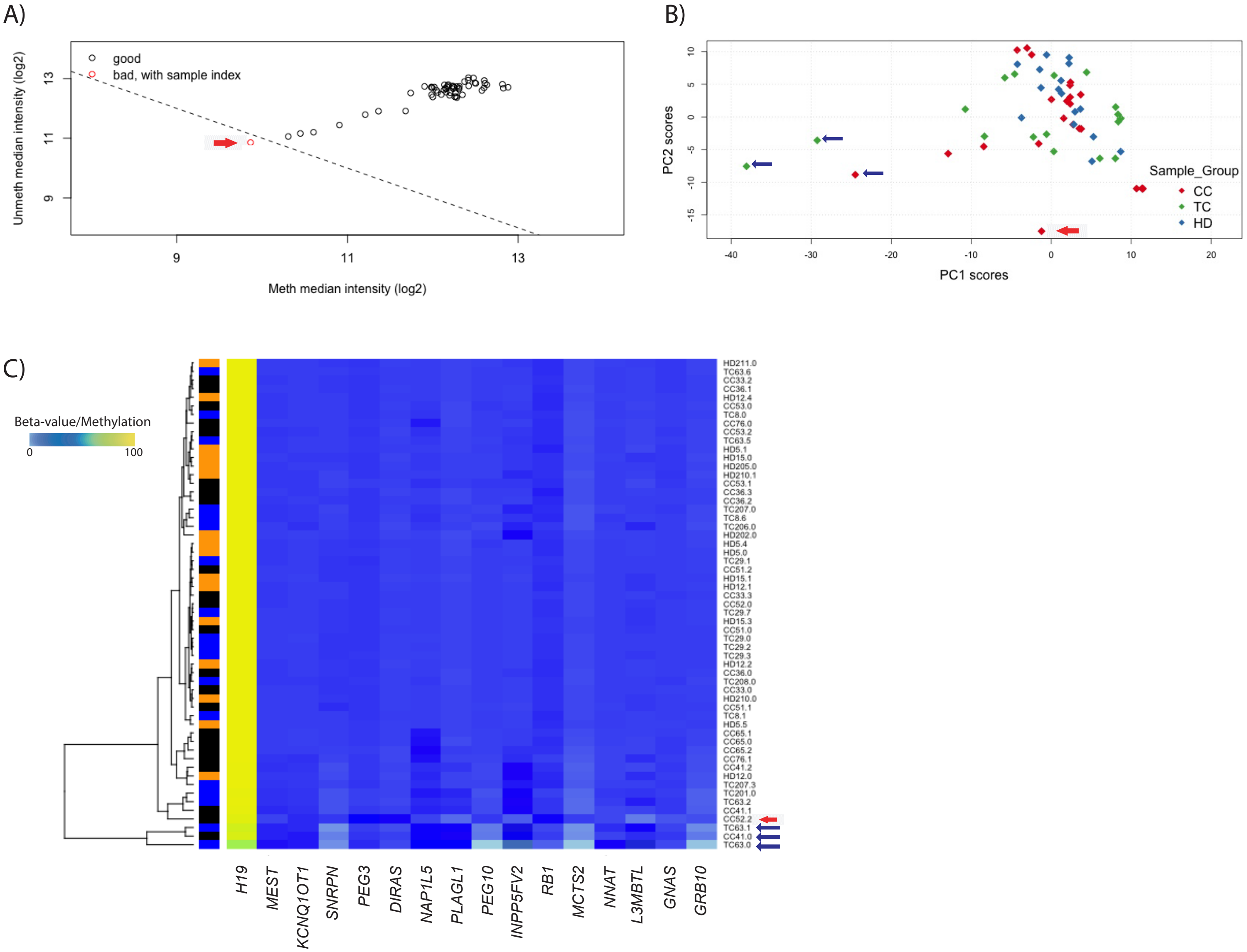

Supp. Figure S7.

A) Quality control plot (minifi) of all samples using the log median of probe intensities in both the methylated and unmethylated channels (pre-normalization) from all samples. Failed samples tend to separate and have lower median intensities. Red arrow points to one sample (CC52-2) that failed QC. B) Principal component analysis (PCA, shinyMethyl) based on normalized beta-values. The sample failing QC, appears to be an outlier of PC2 (red arrow). Three other samples (blue arrows) appear to be outliers in PC1 (one CC sample and two TC samples). C) Hierarchal clustering of the average beta-value/methylation at the imprinting control regions of imprinted gene loci. Arrows indicate samples that were found to not pass quality control (red) and outliers from PCA (blue) from Supp. Fig1B.
